# Impact of Uncertainties in Exposure Assessment on Thyroid Cancer Risk among Persons in Belarus Exposed as Children or Adolescents Due to the Chernobyl Accident

**DOI:** 10.1371/journal.pone.0139826

**Published:** 2015-10-14

**Authors:** Mark P. Little, Deukwoo Kwon, Lydia B. Zablotska, Alina V. Brenner, Elizabeth K. Cahoon, Alexander V. Rozhko, Olga N. Polyanskaya, Victor F. Minenko, Ivan Golovanov, André Bouville, Vladimir Drozdovitch

**Affiliations:** 1 Radiation Epidemiology Branch, Division of Cancer Epidemiology and Genetics, National Cancer Institute, National Institutes of Health, Department of Health and Human Services, Bethesda, Maryland, United States of America; 2 Sylvester Comprehensive Cancer Center, University of Miami, Miami, Florida, United States of America; 3 Department of Epidemiology and Biostatistics, University of California San Francisco, San Francisco, California, United States of America; 4 The Republican Research Center for Radiation Medicine and Human Ecology, Gomel 246040, Belarus; 5 Research Institute for Nuclear Problems, Minsk, Belarus; 6 Burnasyan Federal Medical Biophysical Center, Moscow, Russian Federation; Institute for Health & the Environment, UNITED STATES

## Abstract

**Background:**

The excess incidence of thyroid cancer in Ukraine and Belarus observed a few years after the Chernobyl accident is considered to be largely the result of ^131^I released from the reactor. Although the Belarus thyroid cancer prevalence data has been previously analyzed, no account was taken of dose measurement error.

**Methods:**

We examined dose-response patterns in a thyroid screening prevalence cohort of 11,732 persons aged under 18 at the time of the accident, diagnosed during 1996–2004, who had direct thyroid ^131^I activity measurement, and were resident in the most radio-actively contaminated regions of Belarus. Three methods of dose-error correction (regression calibration, Monte Carlo maximum likelihood, Bayesian Markov Chain Monte Carlo) were applied.

**Results:**

There was a statistically significant (*p*<0.001) increasing dose-response for prevalent thyroid cancer, irrespective of regression-adjustment method used. Without adjustment for dose errors the excess odds ratio was 1.51 Gy^−^ (95% CI 0.53, 3.86), which was reduced by 13% when regression-calibration adjustment was used, 1.31 Gy^−^ (95% CI 0.47, 3.31). A Monte Carlo maximum likelihood method yielded an excess odds ratio of 1.48 Gy^−^ (95% CI 0.53, 3.87), about 2% lower than the unadjusted analysis. The Bayesian method yielded a maximum posterior excess odds ratio of 1.16 Gy^−^ (95% BCI 0.20, 4.32), 23% lower than the unadjusted analysis. There were borderline significant (*p* = 0.053–0.078) indications of downward curvature in the dose response, depending on the adjustment methods used. There were also borderline significant (*p* = 0.102) modifying effects of gender on the radiation dose trend, but no significant modifying effects of age at time of accident, or age at screening as modifiers of dose response (*p*>0.2).

**Conclusions:**

In summary, the relatively small contribution of unshared classical dose error in the current study results in comparatively modest effects on the regression parameters.

## Introduction

The excess incidence of thyroid cancer in Ukraine and Belarus observed a few years after the Chernobyl accident is considered to be largely the result of ^131^I released from the reactor [1], and the excess is particularly marked among those exposed in childhood [2–4]. Thyroid cancer was also increased in some exposed groups of cleanup workers, but the increase was mostly attributed to their residential exposure to ^131^I [5].

The U.S. National Cancer Institute, in collaboration with various external groups of researchers, initiated two cohort screening studies of children and adolescents exposed to Chernobyl fallout in Ukraine and Belarus to better understand the long-term health effects of exposure to radioactive iodines. There have been a number of analyses of these cohorts [3, 4, 6], which document the significantly increased risk of thyroid cancer in relation to ^131^I thyroid dose. Estimation of low dose and low dose-rate risk entails extrapolation of risks from groups exposed at much higher doses and dose rates. Systematic and random dosimetric errors have a major impact on this extrapolation [7]. So-called classical errors are the most serious of these, and generally result in a bias towards the null of the dose-response trend, whereas Berkson errors generally do not result in biased estimates of the dose-response trend for models with Normal error structure [7]. However, Berkson errors would be expected to increase the standard errors of effect estimates [7–9]; as emphasized by Stram *et al*. [9] the inflation in the uncertainties in parameter estimates when the error is purely Berkson is the case irrespective of whether errors are shared or unshared. In the Japanese atomic bomb survivors dose errors are thought to be a mixture of classical and Berkson in form [10, 11], whereas more purely Berkson errors are thought to dominate the dose uncertainties in medical studies [12], and in many occupational ones [9]. Both classical and Berkson errors can include both a shared component, common to all individuals within a group, and an unshared part, unique to an individual within a cohort. Stram *et al*. [9] and Simon *et al*. [13] have proposed different methods for dealing with complex dosimetry systems entailing a complex mixture of shared and unshared errors. Uncertainties in doses accounting for shared and unshared errors have previously been estimated in Chernobyl-exposed groups [14, 15], and in some other groups [13, 16]. Regression calibration, whereby the dose estimate in any regression is replaced by the expected true dose given the measured dose estimate, works well as an error correction method when dose errors are modest [7]. Full-likelihood methods may be indicated when dose errors are more substantial. There are two types of full-likelihood method, namely Monte Carlo likelihood integration (MCML) [17–19], and Bayesian Markov Chain Monte Carlo (MCMC) [10, 20]. Both these full-likelihood methods jointly model disease in terms of the “true” dose; in the case of MCML the profile likelihood, of the true dose conditional on the observed dose is considered, and maximized in the usual way [17, 18].

Prevalent thyroid cancers in the Belarus-US screening cohort were previously analyzed [4] using individual deterministic dose estimates [21]. Stochastic dose estimates have recently become available [22], providing distributions of possible dose estimates for each study subject, in particular taking full account of shared and unshared errors. In this paper we compare regressions of dose on thyroid cancer prevalence using the stochastic thyroid dose estimates with those in the study of Zablotska *et al*. [4] based on the deterministic doses [21], and assess the impact of these changes as well as of adjustment for the effects of dose uncertainty using standard regression-calibration, MCML and Bayesian MCMC procedures on the thyroid cancer risk.

## Data and Methods

### Study data

The Belarusian cohort includes 11,732 individuals who were less than 18 years old on April 26 1986 and were screened for thyroid cancer and other thyroid diseases in 1996–2004. The original individual thyroid dose estimates are based on the thyroid activity measurements made within 2 months after the accident by means of several types of radiation detectors held against the neck, from which the ^131^I activity in the thyroid gland was derived; additional information for dose estimation came from interview data on diet and location during the relevant period and environmental radiation activity measurements [21, 22]. There were exclusions because of incorrect ID (*n* = 20), ineligible age (*n* = 114), poor quality of direct thyroid measurements (*n* = 90); and subjects were not interviewed (*n* = 14). We also excluded from the analysis 121 persons with prior surgery for benign thyroid disease, aplasia or prior thyroid cancer, leaving study population of N = 11,611 study subjects. There were a total of 87 prevalent thyroid cancer cases identified at screening in this subset, exactly as in the data of Zablotska *et al*. [4].

### Deterministic and stochastic thyroid dose estimates

The calculation of the thyroid dose due to intakes of ^131^I includes the consideration of several exposure pathways: (1) ingestion of contaminated milk, which is usually the most important pathway, (2) the ingestion of contaminated milk products, (3) the ingestion of contaminated leafy vegetables, and (4) inhalation of contaminated ground-level air. Fifty-nine parameters are involved in the dose calculation.

For the calculation of the deterministic thyroid dose, each of the 59 parameters is assigned a single value (which may or may not vary from one individual to another), so that only one dose value (called deterministic) is obtained for each individual. For the entire cohort, the deterministic thyroid doses due to intakes of ^131^I were found to range from 0.54 mGy to 33 Gy, with an arithmetic mean of 0.58 Gy [21]. Further details on the basic assumptions and procedures used for the deterministic dose calculations are given in Appendix A and in the paper of Drozdovitch *et al*. [22].

For the calculation of the probabilistic thyroid dose, probability distributions were assigned to parameters involved in the calculation. A Monte-Carlo simulation procedure was then used to obtain the distribution of the stochastic thyroid doses [22]. This procedure is similar to and generally consistent with the 2-dimensional (2D) Monte Carlo method [13]. For a specific dose realization some of the model parameter values were in common among members of subgroups, i.e., *shared* among subjects of those groups, implying that any error made in this parameter was shared by all subjects to whom it applied. Twenty subject-independent, or *shared*, parameters were identified in the dose calculation procedures used in our study. They are parameters of the ecological model that describe the temporal variation of the ground, air, and foodstuff contamination with ^131^I and ^131^I activity in the thyroid gland. Other uncertainties were considered to be subject-dependent, or *unshared*. Twenty eight parameters used for dose calculations were considered to be unshared errors related to measurements of ^131^I activity in thyroid (7 parameters), thyroid mass (1 parameter), biokinetic models of iodine in human body (4 parameters), and imprecise responses to questions administered during the personal interview (16 parameters). Eleven parameters of the dose calculation procedures were considered to be known precisely. These include the radioactive decay constant of ^131^I, energy per decay of ^131^I absorbed in thyroid, fraction of ingested ^131^I transferred to blood, and delay between milking and consumption of seven types of milk and milk products as well as between harvesting and consumption of leafy vegetables. Further details on the general form of the procedures used are given in Appendix A and in the paper of Drozdovitch *et al*. [22].

One thousand sets of stochastic thyroid doses from ^131^I intake were calculated for the the 11,732 Belarusian cohort members [22]. The global arithmetic mean of individual mean thyroid doses for the entire cohort was 0.68 Gy. The arithmetic mean of 1,000 individual stochastic thyroid doses ranged from 0.54 mGy to 39 Gy. The uncertainties in thyroid dose were driven by the unshared errors associated with the estimates of values of the ^131^I activity in the thyroid and thyroid mass of the subject; the contribution of shared errors to the overall uncertainty was small. The geometric standard deviation of stochastic doses varied among cohort members from 1.33 to 5.12 with an arithmetic mean of 1.76 and a geometric mean of 1.73. The largest geometric standard deviations (GSD) were associated with small ^131^I activity in the thyroid. It is acknowledged that the uncertainty associated with the instrumental thyroid dose value may have been grossly underestimated, if there are reasons to believe that: (1) the person that was interviewed was not the study subject with the assigned direct thyroid measurement, or (2) experimental or clerical errors were made during the direct thyroid measurement or its processing. However, in order to assess the doses to all study subjects in the same manner, it was assumed that the direct thyroid measurement was correctly performed and processed, an assumption justified by the very small number of exclusions because of poor quality of direct thyroid measurements (*n* = 90).

The *arithmetic mean* of 1,000 individual *stochastic* dose estimates [22] and the *deterministic* dose estimates [21] used by Zablotska *et al*. [4] are essentially the same for most study subjects (see Fig 4 in Drozdovitch *et al*. [22]). There are however small differences that are for a large part due to the asymmetric nature of log-normal distribution of *individual stochastic* doses, and for a small part to differences in the manner in which the two sets of doses were calculated, such as:

- The way in which the background due to the external and internal contamination of the human body to the signal recorded by the radiation detector used in the direct thyroid measurement was taken into account;
- The uncertainty assigned to the date of measurement, which influences the ecological activity of ^131^I in the thyroid at the time of measurement and, consequently, the ratio of measured to ecological ^131^I activity that defines the *instrumental* thyroid dose; and
- The imputation of foodstuff consumption rates for study subjects with pure memory recall.

### Dose error model

The dosimetry estimation system has a stochastic design to model shared errors, and to account for uncertain dose-related parameters. Using that system, we produced 1,000 simulations of the posterior distribution of thyroid dose for all study subjects. Regression calibration proceeded by using the average of these 1,000 individual stochastic dose realizations, rather than the (deterministic) central estimate, in the regressions on dose. As is standard, for the regression-calibration dose error adjustment we used the arithmetic mean of the sample of 1000 stochastic dose realizations for each individual (which approximates the expectation of the true dose for each individual conditional on all dosimetry information and other parameters) as the plug-in estimate of the true doses. The profile likelihood was derived by integrating the likelihood over these 1,000 dose simulations. Whether using regression calibration (arithmetic mean doses) or MCML, parameter estimates were obtained via maximum likelihood, and profile likelihood confidence bounds derived in the usual way [23]. The Bayesian MCMC method is slightly different, and is described in more detail in Appendix B. In contrast to regression calibration, both Bayesian MCMC and MCML methods entail use of 1,000 realizations of the entire cohort dose rather than 1,000 individual stochastic dose realizations for each cohort member. It should be emphasized that these cohort dose realizations are not independent between individuals. Because of the rather different nature of the model, we report the deviance information criterion (DIC) [24] in Table 1 for the fits of the Bayesian model. In order to assess chain convergence we also estimated the Gelman-Rubin statistic [25], otherwise known as the potential scale reduction factor (PSRF), derived as the square root of the posterior marginal variance to the within-chain variance for each parameter when multiple chains are used; this will be close to 1 if the multiple chains are close to convergence.

**Table 1:** Parameter estimates and 95% profile likelihood-based confidence intervals (95% CI) (or 95% Bayesian credible intervals (BCI)) for analysis of curvature in fits of excess odds ratio model (1) with or without adjustment for dose errors using regression calibration, for various sets of doses. All models have underlying rates adjusted for age (treated categorically), gender and oblast.

Regression model/dose used	Dose-response model	Deviance/deviance information criterion [24]	Df	*p*-value^a^	*α* (/Gy) (95% CI)	*γ* (/Gy) (95% CI)	*κ* (/year of age at exposure) (95% CI)	*τ* (/year of age at screening) (95% CI)	*η* (95% CI)
Original deterministic [4, 21]									
Deterministic	-	1013.135	11601	-					
Deterministic	*αD*	992.264	11600	<0.001	1.51 (0.53, 3.86)	-	-	-	-
Deterministic	*αD* exp[*γD*]	988.299	11599	0.046	3.11 (0.96, 9.98)	-0.15 (-0.37, 0.00)	-	-	-
Deterministic	*αD* exp[*γD + κ*(*e*—8)]	987.499	11598	0.371	3.46 (0.99, 12.09)	-0.16 (-0.37, -0.01)	-0.07 (-0.25, 0.08)	-	-
Deterministic	*αD* exp[*γD + τ*(*a*—22)]	987.211	11598	0.297^b^	3.22 (0.85, 11.38)	-0.16 (-0.37, -0.01)	-	-0.08 (-0.27, 0.07)	-
Deterministic	*αD* exp[*γD + η*1_sex = male_]	984.821	11598	0.062^b^	1.57 (0.28, 5.88)	-0.15 (-0.36, -0.01)	-	-	1.77 (-0.09, 4.44)
Revised doses, using regression calibration (average of 1000 stochastic doses) [22]									
Regression calibration	*αD*	990.875	11600	<0.001^c^	1.31 (0.47, 3.31)	-	-	-	-
Regression calibration	*αD* exp[*γD*]	987.247	11599	0.057	2.52 (0.80, 7.77)	-0.11 (-0.29, 0.00)	-	-	-
Regression calibration	*αD* exp[*γD + κ*(*e*—8)]	986.498	11598	0.387	2.78 (0.81, 9.35)	-0.12 (-0.29, -0.01)	-0.07 (-0.24, 0.08)	-	-
Regression calibration	*αD* exp[*γD + τ*(*a*—22)]	986.194	11598	0.305^b^	2.61 (0.71, 8.83)	-0.12 (-0.29, -0.01)	-	-0.08 (-0.27, 0.07)	-
Regression calibration	*αD* exp[*γD + η*1_sex = male_]	984.573	11598	0.102^b^	1.45 (0.30, 5.16)	-0.11 (-0.28, -0.00)	-	-	1.50 (-0.29, 3.90)
Revised doses, using full likelihood methods (1000 stochastic doses) [22]									
MCML	*αD*	989.804	11600	<0.001^c^	1.48 (0.53, 3.87)	-	-	-	-
MCML	*αD* exp[*γD*]	986.693	11599	0.078	2.79 (0.83, 9.05)	-0.10 (-0.29, 0.01)	-	-	-
MCMC	*αD*	1012.6^d^	11600	-	1.16^e^ (0.20, 4.32)^f^	-	-	-	-
MCMC	*αD* exp[*γD*]	1024.7^d^	11599	-	2.05^e^ (0.21, 15.20)^f^	-0.13 (-0.33, -0.00)^e^	-	-	-

^a^unless otherwise stated all *p*-values refer to the improvement in fit of the current row in the Table with that of the model fitted in the row immediately above.

^b^ *p*-value of improvement in fit compared with a model with linear-exponential dose terms.

^c^ *p*-value of improvement in fit compared with a model with no dose terms.

^d^deviance information criterion [24].

^e^posterior distribution maximum probability estimate.

^f^95% Bayesian credibility interval (BCI).

### Thyroid cancer risk model

The primary statistical model used was a logistic model of the odds ratio (OR), in which the probability of subject *i* with age at screening *a*, gender *s*, age at exposure *e* at the time of the accident (1986) and with true thyroid dose *D*, being a case of thyroid cancer is given by:

$$\frac{\text{exp}\left[\beta_{0}+\beta_{s}1_{sex=male}+\sum_{m=1}^{N_{o}}\beta_{mo}1_{oblast=k}+\sum_{k=1}^{N_{a}}\beta_{ka}1_{a_{k-1}\leq a<a_{k}}\right]\left[1+\alpha D\text{exp}\left(\gamma D+\kappa [e-8]+\tau [a-22]+\eta 1_{sex=male}\right)\right]}{1+\text{exp}\left[\beta_{0}+\beta_{s}1_{sex=male}+\sum_{m=1}^{N_{o}}\beta_{mo}1_{oblast=k}+\sum_{k=1}^{N_{a}}\beta_{ka}1_{a_{k-1}\leq a<a_{k}}\right]\left[1+\alpha D\text{exp}\left(\gamma D+\kappa [e-8]+\tau [a-22]+\eta 1_{sex=male}\right)\right]}$$ (1)

[The age at exposure, *e*, and age at screening, *a*, are approximately centered by subtracting off their approximate mean values in the data, namely 8 and 22 years, respectively; this facilitates convergence of the iteratively-reweighted least squares algorithm used to maximize the likelihood [23].]

### Ethics Statement

The data were hosted at three collaborating institutions: Republican Research Center for Radiation Medicine and Human Ecology, Gomel, Belarus, Columbia University/University of California San Francisco (UCSF), and the National Cancer Institute (NCI). All subjects signed an informed consent form, and the study was reviewed and approved by the institutional review boards of the participating institutions in both Belarus (Institutional Review Board of Republican Research Center for Radiation Medicine and Human Ecology, Gomel) and the United States (Special Studies Institutional Review Board of the National Cancer Institute). We obtained written informed consent from the next of kin, caretakers, or guardians on behalf of the minors/children enrolled in the study. The institutional review boards in both countries (Belarus, USA) approved this consent procedure. The data were de-identified before transfer to the United States participating institutions. The key to the data exists in Belarus, but US researchers did not have access to it at any point. Anonymized data can be provided upon request with conditions agreeable to the three parties (Republican Research Center for Radiation Medicine and Human Ecology, Gomel, Belarus, Columbia University/UCSF, NCI). At NCI, it has to be formalized through the Technical Transfer Center. This study involved human subjects and to protect the privacy of study participants, data requests will be reviewed by the NCI DCEG Data Repository Committee. Requests should be directed to NCIDCEGDataAccessRequests@mail.nih.gov.

## Results

### Comparison of effects of revision to dosimetry

**Fig 1:**
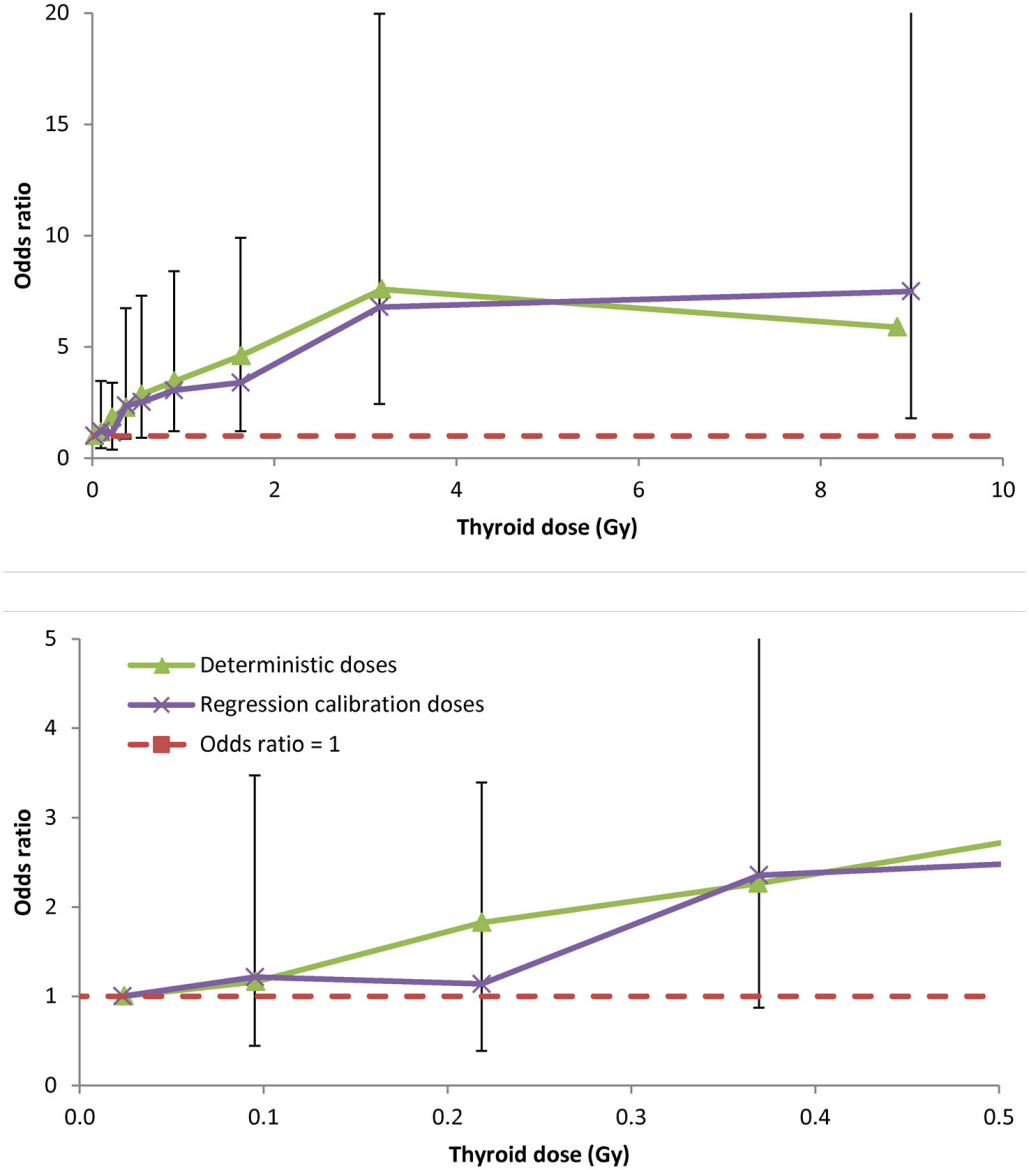
Dose-response (+95 CI) for thyroid cancer in relation to deterministic [4, 21], and regression-calibration adjusted doses (arithmetic means of 1,000 individual stochastic doses) [22]. The models are adjusted for age (treated categorically), gender and oblast in the baseline. Dashed red line shows odds ratio = 1. The lower panel shows the lower dose (<0.5 Gy) part of the dose response.

Table 1 demonstrates that using a simple linear link in the logistic model (1), there is a highly statistically significant increasing dose-response (*p*<0.001) for all sets of dose estimates. The dose-response using the various sets of dose estimates is shown in Fig 1.

### Comparison of effects of various adjustments for dose error in logistic model

**Fig 2:**
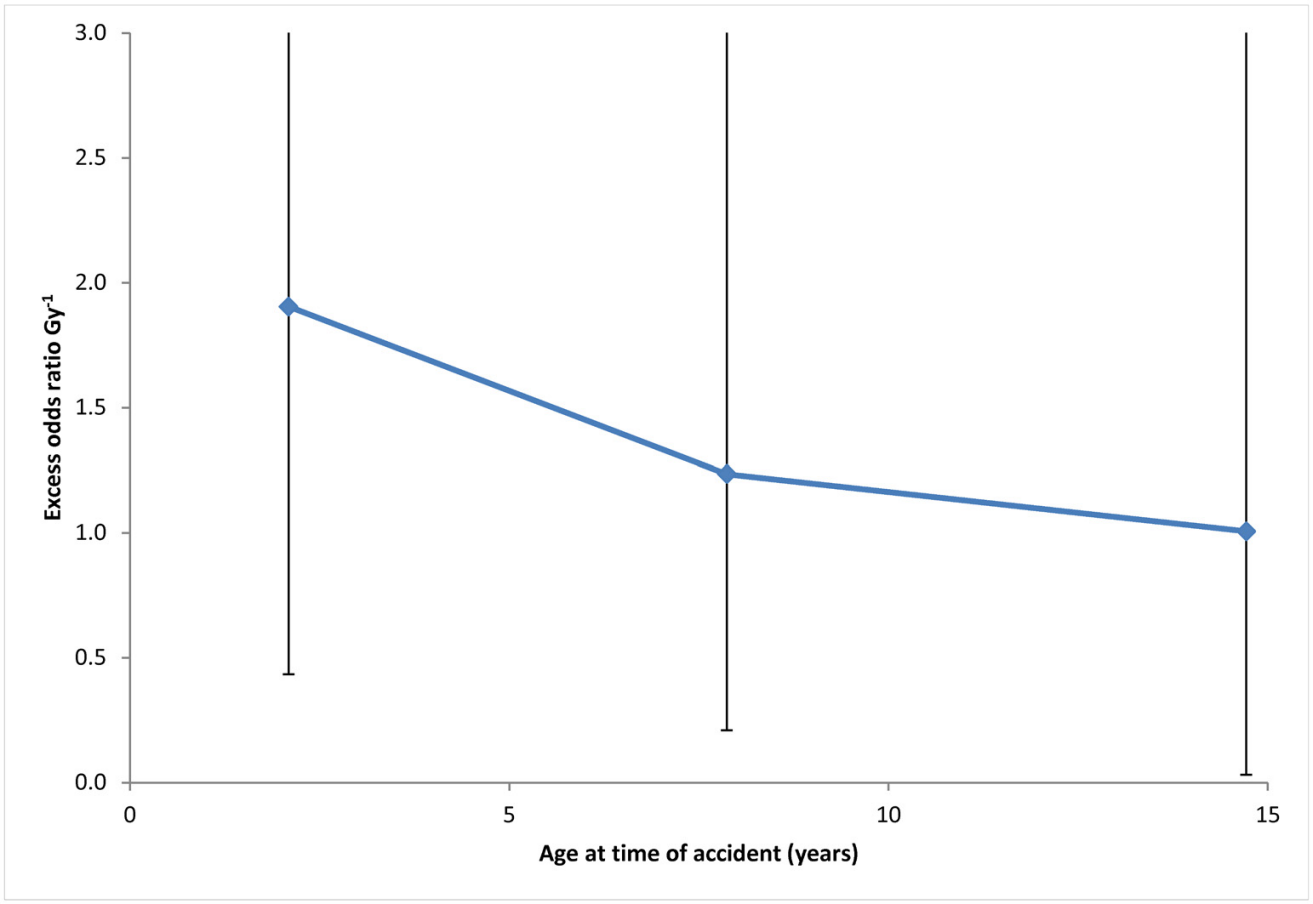
Variation of excess relative risk with age at the time of the accident (using regression calibration adjusted doses). Other details as for Fig 1.

Table 1 demonstrates that without adjustment for dose errors the excess odds ratio (EOR) is 1.51 Gy^−^ (95% CI 0.53, 3.86), and this is somewhat reduced (by about 13%) if regression-calibration adjustment (arithmetic mean of 1,000 individual stochastic dose estimates) is used, 1.31 Gy^−^ (95% CI 0.47, 3.31). Standard errors are also almost unaffected by adjustment for dose error. A Monte Carlo maximum likelihood method yields an EOR of 1.48 Gy^−^ (95% CI 0.53, 3.87), about 2% lower than the unadjusted dose estimates (Table 1). The Bayesian method yields a maximum posterior EOR of 1.16 Gy^−^ (95% BCI 0.20, 4.32), about 23% lower than the unadjusted dose estimates (Table 1). However, in contrast to regression calibration, there are slightly wider confidence intervals on the parameters. The linear Bayesian model *α* coefficient has PSRF of 1.027, while for the linear-exponential Bayesian models the *α* coefficient has PSRF 1.006, and the *γ* coefficient has PSRF 1.008 –all indicating satisfactory convergence. There were no indications of lack of convergence for any other type of dose error correction model (regression calibration, MCML). Table 1 demonstrates that there are borderline significant (*p* = 0.057–0.078), indications of downward curvature in the dose-response, depending on the adjustment methods used. There are also borderline significant (*p* = 0.102) modifying effects of gender on the EOR, with males having markedly higher radiation risk (Table 1). Table 1 demonstrates that the effects of age at the time of the accident or age at screening as modifiers of the radiation dose-response are generally not statistically significant (*p*>0.2). Fig 2 demonstrates the weak indications of reduction of excess odds ratio with increasing age at the time of the accident.

## Discussion

Re-analysis of the prevalence data from the Belarus-US thyroid screening study, and using the set of stochastic dose estimates, demonstrates that there is a highly statistically significant increasing dose-response (*p*<0.001), confirming the results of an earlier analysis of this dataset [4]. The use of arithmetic mean (regression calibration) *vs* deterministic thyroid dose estimates resulted in relatively modest differences in regression risk estimates. Adjustment of the regression for dose errors using MCML yielded slight decreases in radiation risk estimates relative to the deterministic estimates, while using regression calibration or MCMC resulted in somewhat more substantial decreases in radiation risk. Adjustment of the regression for dose errors using full-likelihood methods somewhat enlarged parameter confidence intervals.

It is generally recognized that dose measurement error can alter substantially the shape of the radiation dose-response curve [26], which is crucial to the extrapolation of risks at high dose and high dose-rates to those at low/moderate doses and low dose-rates. Regression calibration [7], entailing the substitution of the “estimated dose” by the expectation of the “true dose” given the estimated one, has been much used in the Japanese atomic bomb survivor data [27–32]. As emphasized by Carroll *et al*. [7], this is an approximate method in non-linear dose-effect relationships, leading to reasonable adjusted point estimates of the model parameters when errors are not too large, but does not fully take account of all the variability induced by the measurement errors. Various full-likelihood methods, in particular Bayesian MCMC and MCML methods are more appropriate when errors are larger. An adapted Bayesian method of correction for measurement error—the two-stage Bayesian method—has been applied to the fitting of generalized relative risk models to the Japanese atomic bomb survivor cancer mortality data [10, 33, 34]. The Bayesian approach used in this paper is somewhat different from the above Bayesian approaches since the proposed approach is based on Bayesian model averaging rather than the measurement-error model approach used above. It is described at further length in Appendix B. The findings of a similar degree of adjustment when regression-calibration and various full-likelihood methods are used parallels those in many other radiation datasets [10, 17, 35, 36].

A remarkable feature of the present analysis is the minimal effect of adjustment for dose error on the thyroid cancer risk estimates. The MCML method leads to a 2% decrease in EOR, regression calibration results in a 13% decrease in EOR, while the Bayesian MCMC method yields a 23% decrease. In the light of the overall uncertainties in risk estimates we do not judge that these relatively modest differences are of any significance. All three methods take account of mixed Berkson and classical errors in dose, arising from the distinct measurement and estimation associated with thyroid mass and ^131^I thyroid activity measurements. Almost all uncertainty is defined by unshared (classical) errors associated with errors in activity of ^131^I in the thyroid and thyroid mass. These results parallel those in the independent analysis of the Ukraine thyroid prevalence data [36], in which two different types of regression calibration and a Monte Carlo maximum likelihood method were employed to correct for potential effects of dose error, none of which made appreciable difference on the dose response trend parameters. Dose uncertainties are generally larger than those in the parallel Ukrainian cohort [36]. However, the reasons for the relatively modest impact of adjusting for dose error are largely a consequence of the fact that these errors are still relatively small. The uncertainty has a geometric mean GSD of 1.73 (vs 1.47 in Ukraine) and a higher fraction of *stochastic* thyroid doses with a GSD greater than 2.0 (11% in this study vs 3.8% in Ukraine) [22]. An interesting (and reassuring feature) of the present analysis, as with the analysis of the Ukraine thyroid prevalence data [36], is that regression calibration gives similar results to either full-likelihood method (MCML, Bayesian MCMC), although parameter confidence intervals are somewhat wider with either of the full-likelihood methods (Table 1).

It should be emphasized that the study is completely separate from the parallel study in Ukraine, and the dosimetric reconstruction is also of very different form, as we make clear in the Methods and above. Given the differences between the two datasets, a joint analysis is not indicated.

The prevalence excess odds ratio that we derive of 1.31 Gy^−^ (95% CI 0.47, 3.31) using the regression-calibration method (Table 1) is somewhat lower than, but statistically consistent with (*p* = 0.12) that which can be derived from the Japanese atomic bomb survivors exposed to external radiation under the age of 20, 3.07 Gy^−^ (90% CI 2.14, 4.14) [37]. It is lower than (and marginally statistically incompatible with (*p* = 0.04)) the estimate of 7.7 Gy^−^ (95% CI 2.1, 28.7) derived from a pooled analysis of five childhood-exposed groups [38]. However, the analyses of United Nations Scientific Committee on the Effects of Atomic Radiation (UNSCEAR) [37] and Ron *et al*. [38] are based on incidence data, and the interpretation is therefore somewhat different from the prevalence risk that we estimate. Ron *et al*. [38] also computed a pooled excess relative risk (ERR) /Gy allowing for a non-zero ERR at zero dose (essentially allowing for an additional offset in risk independent of radiation dose), which was 3.8 Gy^−^ (95% CI 1.4, 10.7). Importantly, pooled risks in the study of Ron *et al*. [38] are estimated for populations exposed to external ionizing radiations (gamma and X-rays), while in our analyses the main component of the radiation dose (95%) was due to internal exposures to ^131^I.

We observed borderline significant indications of downward curvature (in other words, a progressive reduction with increasing dose in the upward slope of ERR, rather than negative slope) in the dose-response (*p* = 0.057–0.078, Table 1), consistent with the previous findings in this cohort of Zablotska *et al*. [4]. The thyroid is known to be one of the most radiosensitive organs with regard to cancer risks [37], in particular there is abundant literature documenting excess thyroid cancer after exposure to external radiation in childhood [38]. The pooled analysis of Ron *et al*. [38] indicated that in general thyroid cancer exhibited a linear dose-response, with indications of a reduction of risk at high doses (>20 Gy). However, the analyses of Ron *et al*. [38] were all of thyroid cancer incidence, and so may not be directly comparable with the prevalence analysis conducted here. More recently Sigurdson *et al*. [39] and Veiga *et al*. [40] similarly observed a reduction in the thyroid cancer dose-response, although at a much higher dose, of about 20 Gy, in studies of patients treated with radiotherapy for first primary cancer in childhood. A recent update of solid cancer incidence in the LSS cohort indicated possible flattening of the dose response at doses of about 2 Gy or more [41]. Cardis *et al*. also observed a turnover in dose-response above about 5 Gy in a case-control study of Chernobyl-exposed children in Belarus and the Russian Federation [42]. As such, the turnover that we, Zablotska *et al*. [4] and Cardis *et al*. [42] observe, and at a somewhat lower dose, of less than 5 Gy (Fig 1), is somewhat unusual. Analysis of the prevalent thyroid cancers in Ukraine yielded non-significant indications of downward curvature [3], findings reflected also in analysis adjusting for dose errors using regression calibration or MCML methods (*p* = 0.102–0.112) [36].

There were at most weak indications of reduction of relative risk with increasing time after the accident (Table 1). There are some indications of eventual reductions of thyroid cancer relative risk with increasing time after exposure among those exposed in childhood [38, 43, 44]. It is likely that our cohort, with follow-up confined to a relatively narrow time interval, 1996–2004, about 10–18 years after the Chernobyl accident, lacks the power to detect such downturns in risk, which in any case would not be expected until 15–19 years after the accident [38]. We observed weak and statistically non-significant (*p*>0.2) indications of variations of excess odds ratio with age at screening (Table 1). However, as this variable is very highly correlated with age at exposure the results are difficult to interpret.

Although the general patterns of risk are similar in the Ukraine and Belarus prevalence datasets, the parameter estimates are substantially different. For example the linear EOR coefficient we derive, 1.31 Gy^−^ (95% CI 0.47, 3.31) (Table 1) is somewhat less than that in the Ukraine data, 5.78 Gy^−^ (95% CI 1.92, 27.04) [36]. There are equally substantial differences in the linear coefficients if linear-exponential models are fitted, 2.52 Gy^−^ (95% CI 0.80, 7.77) (Table 1) compared with 9.72 Gy^−^ (95% CI 2.67, 94.31) in the Ukraine data [36], although the exponential dose coefficients are more similar, -0.11 Gy^−^ (95% CI -0.29, 0.00) (Table 1) compared with -0.10 Gy^−^ (95% CI -0.28, 0.02). It is possible that some of this difference reflects the much better estimates of thyroid mass in the Belarus data, which are based on individual estimates of thyroid volume [21], in contrast to the grouped estimates used in Ukraine [36].

There are certain limitations in the dosimetric evaluation used here, which are developed at greater length in the paper of Drozdovitch *et al*. [22]. The dose estimates for each individual are largely based on interview, with the person or with a surrogate, to determine patterns of behavior in the short period after the accident. Drozdovitch *et al*. assume that the variation with time of the ecological estimate of thyroid dose, $Q_{t}^{ecol}(t)$ (see Appendix A); as they make clear, this is likely to depend crucially on the agreement between the instrumental and ecological dose estimates, in turn a function of the accuracy of recall of patterns of behavior of the study subjects [22]. Work is underway to verify this. The thyroid mass estimates used in the study were derived from age-specific estimates made in a group of individuals in 1991–1996 who were not part of the cohort. Although there is no reason to suspect that these measurements are appreciably biased estimates of those that would have obtained in the cohort at the time of the accident (1986) [22], this does introduce some uncertainty in the thyroid doses, which is not taken into account in the stochastic dosimetry, nor therefore in our analysis.

## Conclusions

The results of the paper are based on a screening study of the most heavily exposed populations in Belarus who were under 18 years of age at the time of the Chernobyl accident. The paper updates previous analyses [4] by using stochastic rather than deterministic thyroid dose estimates. In addition, this paper addresses for the first time the errors that are present in absorbed thyroid doses, and their effect on thyroid cancer risk estimates in the Belarus-US screening cohort. The effects of adjusting for dose error are minimal, resulting in changes to thyroid cancer risk estimates of -23% to -2%. Effects on parameter standard errors are also minimal, although the two full-likelihood methods both yield somewhat wider confidence intervals. The relatively modest changes in risk resulting from taking dose errors into account is largely a consequence of the modest size of the errors. There are borderline significant reductions in the upward slope of thyroid cancer risk at high doses, and borderline significant adjustments to the dose-response for gender, with males at appreciably higher risk, although this finding is not statistically significant. Given that none of the three dose-error correction methods had decisive advantages, it may be wise to consider the totality of the ranges of error-corrected estimates rather than to rely on the range from a single technique.

## Appendix A. Description of deterministic and stochastic dose estimation

### Deterministic dose estimates

The deterministic thyroid doses were estimated using input data specific to each cohort member (direct thyroid measurement and personal interview) and ecological data (e.g., ^131^I ground deposition in the settlements where any of the cohort members resided). Ecological and biokinetic models were used to reconstruct the transport of ^131^I from the ground deposition to the child’s thyroid via the activity intake with contaminated air and foodstuffs calculated using personal interview data on individual behavior and consumption rates of foodstuffs. For each study subject *k*, two estimates of thyroid dose, differing in the manner in which the activity of ^131^I in the thyroid was assessed, were calculated:

1. an ‘‘instrumental” thyroid dose, *D*_*k*_, based on the measured ^131^I activity in the thyroid at time *t*_*m*_ after the accident, $Q_{k}^{meas}(t_{m})$, which is derived from the direct thyroid measurement; and
2. an ‘‘ecological” thyroid dose, in which the ^131^I activity in the thyroid, $Q_{k}^{ecol}(t)$, is calculated for any time *t* after the accident using ecological and biokinetic models, together with personal interview data on individual behavior and consumption rates of foodstuffs.

The ‘‘instrumental” thyroid dose estimate, being based on a dose-related measurement performed on each study subject, is better than the ‘‘ecological” dose estimate and is recommended for use in the assessment of radiation risks. The main purpose of the ‘‘ecological” dose estimate is to provide an evaluation of the reliability of the ‘‘instrumental” thyroid dose estimate. The ‘‘ecological” thyroid dose ($D_{k}^{ecol}$, mGy) for subject *k* is calculated as follows:

$$D_{k}^{ecol}=\frac{U_{c}E_{th}}{m_{k}}\int_{0}^{T}Q_{k}^{ecol}(t)dt$$ (2)

where *U*_*c*_ is a unit conversion factor of 13.82 (Bq kBq^–^ g kg^–^ J MeV^–^ s d^–^ mGy Gy^−^), *m*_*k*_ is the subject-specific mass of the thyroid (g), *E*_*th*_ is the mean energy absorbed in the thyroid per decay of ^131^I in the thyroid (MeV decay^–^), and $Q_{k}^{ecol}(t)$ is the ‘‘ecological” activity of ^131^I in the thyroid of study subject *k* at time *t* (kBq). The period of integration is from the time of the accident on 26 April 1986 (*t* = 0) until 30 June 1986 (*t* = *T* = 66 days).

To calculate the ‘‘instrumental” thyroid dose (*D*_*k*_, mGy), the calculated ‘‘ecological” ^131^I activity in the thyroid at time *t*_*m*_, $Q_{k}^{ecol}(t_{m})$, is replaced in expression (A1) with the measured activity, $Q_{k}^{meas}(t_{m})$, and it is assumed that the relative shape of the variation of $Q_{k}^{ecol}(t)$ with time is correct, so that the adjustment at time *t*_*m*_ also applies to any other time after the accident. Under those conditions, one obtains:

$$D_{k}=\frac{Q_{k}^{meas}(t_{m})}{Q_{k}^{ecol}(t_{m})}D_{k}^{ecol}$$ (3)

A more detailed description of the methodology used to calculate the ‘‘instrumental” thyroid doses for all members of the cohort of Belarusian children can be found elsewhere [21].

### Stochastic dose estimates: general 2D Monte Carlo approach

Using Monte Carlo simulation to estimate the uncertainties in instrumental thyroid doses, we calculated 1,000 sets of cohort instrumental thyroid doses, which take into account classification of errors as shared or unshared. This procedure is similar to and generally consistent with the 2-dimensional (2D) Monte Carlo method [13]. For a specific dose realization some of the model parameter values were in common among members of subgroups, i.e., shared among subjects of those groups, implying that any error made on this parameter was shared by all subjects to whom it applied. At the beginning of calculation of each dose set for the entire cohort, we sampled values for all shared parameters from their probability distributions. To calculate one dose set for the entire cohort, the same value for each shared parameter was used for all cohort members for whom this parameter was considered to be shared. This step intentionally introduced correlations in each simulated dose set between individual dose estimates of the study subjects who shared parameter values. In the process of dose set simulation, we sampled values of unshared parameters for each cohort member from their distributions and calculated one dose realization for cohort member *k*, *D*_*i*,*k*_. A set of doses from *D*_i,1_ to *D*_i,11732_ therefore represents stochastic set number *i* of cohort thyroid doses. The thousand realizations of dose, from *D*_1,k_ to *D*_1000,k_, for cohort member *k*, represent the individual stochastic thyroid doses of that cohort member. A detailed description of the evaluation of the errors associated with the parameters used for calculation of thyroid doses is presented elsewhere [22].

## Appendix B. Description of Bayesian Markov Chain Monte Carlo model fitting

We outline a Bayesian MCMC approach to evaluate dose uncertainty. This has the advantage over simpler (e.g., regression calibration) methods that it can consider more complex dosimetry systems and patterns of error, although comparable with other full-likelihood methods such as MCML [17–19] in this respect. The dosimetry system produces a number of realizations of the entire set of doses, that characterize the state of knowledge about doses for this population. Our main goal was to estimate the radiation risk coefficients, *α*,*γ*,*κ*,*τ*,*η*, and their Bayesian credible interval (BCI), accounting for both the usual statistical sampling error and uncertainty in the dosimetry. Our approach can deal with various types of outcomes, such as continuous, time-to-event, and count data. In this application we considered an outcome variable with binary (binomial) error.

In order to perform Bayesian inference we must formulate prior distributions on all model parameters. We assumed normal prior distributions for the parameters in expression (1). Suppose we had *M* dose realizations (*M* = 1,000 here), and let *θ* be the dose vector index variable in the model. The parameter, *θ*, is distributed as a multinomial distribution, *Mult*(1,*π*). The probability vector, *π*, has a hyper-prior distribution given by a Dirichlet distribution, *Dirichlet*(*w*), with *w* = 1, so that dose realizations are chosen with equal probability *a priori*. The MCMC method was then used to produce a sample from the posterior distribution of the parameters of interest. Parameter estimates (the maximum of the posterior density) and their corresponding 95% BCI in Table 1 are based on 20,000 posterior samples after 10,000 burn-in iterations with three chains.

## References

[1] Williams D. Radiation carcinogenesis: lessons from Chernobyl. Oncogene. 2008;27 Suppl 2:S9–18. doi: onc2009349 [pii]; 10.1038/onc.2009.349 19956182

[2] Kazakov VS, Demidchik EP, Astakhova LN. Thyroid cancer after Chernobyl. Nature. 1992;359(6390):21 10.1038/359021a0 1522879

[3] Tronko MD, Howe GR, Bogdanova TI, Bouville AC, Epstein OV, Brill AB, et al A cohort study of thyroid cancer and other thyroid diseases after the Chornobyl accident: thyroid cancer in Ukraine detected during first screening. J Natl Cancer Inst. 2006;98(13):897–903. doi: 98/13/897 [pii]; 10.1093/jnci/djj244 16818853

[4] Zablotska LB, Ron E, Rozhko AV, Hatch M, Polyanskaya ON, Brenner AV, et al Thyroid cancer risk in Belarus among children and adolescents exposed to radioiodine after the Chornobyl accident. Br J Cancer. 2011;104(1):181–7. doi: 6605967 [pii]; 10.1038/sj.bjc.6605967 21102590 PMC3039791

[5] Kesminiene A, Evrard AS, Ivanov VK, Malakhova IV, Kurtinaitise J, Stengrevics A, et al Risk of thyroid cancer among Chernobyl liquidators. Radiat Res. 2012;178(5):425–36. 10.1667/RR2975.1 22998226

[6] Brenner AV, Tronko MD, Hatch M, Bogdanova TI, Oliynik VA, Lubin JH, et al I-131 dose response for incident thyroid cancers in Ukraine related to the Chornobyl accident. Environ Health Perspect. 2011;119(7):933–9. 10.1289/ehp.1002674 21406336 PMC3222994

[7] Carroll RJ, Ruppert D, Stefanski LA, Crainiceanu CM. Measurement error in nonlinear models A modern perspective. Boca Raton, FL: Chapman and Hall/CRC; 2006 p. 1–488.

[8] Bateson TF, Wright JM. Regression calibration for classical exposure measurement error in environmental epidemiology studies using multiple local surrogate exposures. Am J Epidemiol. 2010;172(3):344–52. doi: kwq123 [pii]; 10.1093/aje/kwq123 20573838

[9] Stram DO, Preston DL, Sokolnikov M, Napier B, Kopecky KJ, Boice J, et al Shared dosimetry error in epidemiological dose-response analyses. PLoS ONE. 2015;10(3):e0119418 10.1371/journal.pone.0119418 25799311 PMC4370375

[10] Little MP, Hoel DG, Molitor J, Boice JD Jr, Wakeford R, Muirhead CR. New models for evaluation of radiation-induced lifetime cancer risk and its uncertainty employed in the UNSCEAR 2006 report. Radiat Res. 2008;169(6):660–76. doi: RR1091 [pii]; 10.1667/RR1091.1 18494541

[11] Pierce DA, Kellerer AM. Adjusting for covariate errors with nonparametric assessment of the true covariate distribution. Biometrika. 2004;91(4):863–76. Epub 12/2004. 10.1093/biomet/91.4.863

[12] Schafer DW, Lubin JH, Ron E, Stovall M, Carroll RJ. Thyroid cancer following scalp irradiation: a reanalysis accounting for uncertainty in dosimetry. Biometrics. 2001;57(3):689–97. 11550916

[13] Simon SL, Hoffman FO, Hofer E. The two-dimensional Monte Carlo: a new methodologic paradigm for dose reconstruction for epidemiological studies. Radiat Res. 2015;183(1):27–41. 10.1667/RR13729.1 . 25496314 PMC4423557

[14] Drozdovitch V, Maceika E, Khrouch V, Zvonova I, Vlasov O, Bouville A, et al Uncertainties in individual doses in a case-control study of thyroid cancer after the Chernobyl accident. Radiat Prot Dosimetry. 2007;127(1–4):540–3. doi: ncm360 [pii]; 10.1093/rpd/ncm360 17634207

[15] Drozdovitch V, Khrouch V, Maceika E, Zvonova I, Vlasov O, Bratilova A, et al Reconstruction of radiation doses in a case-control study of thyroid cancer following the Chernobyl accident. Health Phys. 2010;99(1):1–16. 10.1097/HP.0b013e3181c910dd 00004032-201007000-00001 [pii]. 20539120 PMC2885044

[16] Land CE, Kwon D, Hoffman FO, Moroz B, Drozdovitch V, Bouville A, et al Accounting for shared and unshared dosimetric uncertainties in the dose response for ultrasound-detected thyroid nodules after exposure to radioactive fallout. Radiat Res. 2015;183(2):159–73. 10.1667/RR13794.1 . 25574587 PMC4423551

[17] Fearn T, Hill DC, Darby SC. Measurement error in the explanatory variable of a binary regression: regression calibration and integrated conditional likelihood in studies of residential radon and lung cancer. Stat Med. 2008;27(12):2159–76. 10.1002/sim.3163 18081195

[18] Stram DO, Kopecky KJ. Power and uncertainty analysis of epidemiological studies of radiation-related disease risk in which dose estimates are based on a complex dosimetry system: some observations. Radiat Res. 2003;160(4):408–17. doi: 3046 [pii]. 12968933 10.1667/3046

[19] Stayner L, Vrijheid M, Cardis E, Stram DO, Deltour I, Gilbert SJ, et al A Monte Carlo maximum likelihood method for estimating uncertainty arising from shared errors in exposures in epidemiological studies of nuclear workers. Radiat Res. 2007;168(6):757–63. doi: RR0677 [pii]; 10.1667/RR0677.1 18088178

[20] Kwon D, Hoffman FO, Moroz BE, Simon SL. Bayesian dose–response analysis for epidemiological studies with complex uncertainty in dose estimation. Stat Med. 2015: n/a-n/a. 10.1002/sim.6635 26365692

[21] Drozdovitch V, Minenko V, Khrouch V, Leshcheva S, Gavrilin Y, Khrutchinsky A, et al Thyroid dose estimates for a cohort of Belarusian children exposed to radiation from the Chernobyl accident. Radiat Res. 2013;179(5):597–609. 10.1667/RR3153.1 23560632 PMC3682838

[22] Drozdovitch V, Minenko V, Golovanov I, Khrutchinsky A, Kukhta T, Kutsen S, et al Thyroid dose estimates for a cohort of Belarusian children exposed to ^131^I from the Chernobyl accident: assessment of uncertainties. Radiat Res. 2015;184(2):203–18. 26207684 10.1667/rr13791.1 PMC4548301

[23] McCullagh P, Nelder JA. Generalized linear models 2nd edition Monographs on statistics and applied probability 37 Boca Raton, FL: Chapman and Hall/CRC; 1989 p. 1–526.

[24] Spiegelhalter DJ, Best NG, Carlin BP, van der Linde A. Bayesian measures of model complexity and fit. J Royal Statist Soc Series B—Statistical Methodology. 2002;64(4):583–639. Epub 5/2002. 10.1111/1467-9868.00353

[25] Gelman A, Rubin DB. Inference from iterative simulation using multiple sequences. Statist Science. 1992;7(4):457–72.

[26] Thomas D, Stram D, Dwyer J. Exposure measurement error: influence on exposure-disease relationships and methods of correction. Annu Rev Public Health. 1993;14:69–93. 10.1146/annurev.pu.14.050193.000441 8323607

[27] Pierce DA, Stram DO, Vaeth M, Schafer DW. The errors-in-variables problem: considerations provided by radiation dose-response analyses of the A-bomb survivor data. J Am Statist Assoc. 1992;87(418):351–9. 10.1080/01621459.1992.10475214

[28] Pierce DA, Stram DO, Vaeth M. Allowing for random errors in radiation dose estimates for the atomic bomb survivor data. Radiat Res. 1990;123(3):275–84. 2217725

[29] Little MP, Muirhead CR. Evidence for curvilinearity in the cancer incidence dose-response in the Japanese atomic bomb survivors. Int J Radiat Biol. 1996;70(1):83–94. 8691040 10.1080/095530096145364

[30] Little MP, Muirhead CR. Curvilinearity in the dose-response curve for cancer in Japanese atomic bomb survivors. Environ Health Perspect. 1997;105 Suppl 6:1505–9. 9467073 10.1289/ehp.97105s61505 PMC1469947

[31] Little MP, Muirhead CR. Curvature in the cancer mortality dose response in Japanese atomic bomb survivors: absence of evidence of threshold. Int J Radiat Biol. 1998;74(4):471–80. 9798958 10.1080/095530098141348

[32] Little MP, Muirhead CR. Derivation of low-dose extrapolation factors from analysis of curvature in the cancer incidence dose response in Japanese atomic bomb survivors. Int J Radiat Biol. 2000;76(7):939–53. 10923618 10.1080/09553000050050954

[33] Little MP, Deltour I, Richardson S. Projection of cancer risks from the Japanese atomic bomb survivors to the England and Wales population taking into account uncertainty in risk parameters. Radiat Environ Biophys. 2000;39(4):241–52. 11200968 10.1007/s004110000070

[34] Bennett J, Little MP, Richardson S. Flexible dose-response models for Japanese atomic bomb survivor data: Bayesian estimation and prediction of cancer risk. Radiat Environ Biophys. 2004;43(4):233–45. 10.1007/s00411-004-0258-3 15565453

[35] Little MP, Kwon D, Doi K, Simon SL, Preston DL, Doody MM, et al Association of chromosome translocation rate with low dose occupational radiation exposures in U.S. radiologic technologists. Radiat Res. 2014;182(1):1–17. 10.1667/RR13413.1 . 24932535 PMC4829936

[36] Little MP, Kukush AG, Masiuk SV, Shklyar S, Carroll RJ, Lubin JH, et al Impact of uncertainties in exposure assessment on estimates of thyroid cancer risk among Ukrainian children and adolescents exposed from the Chernobyl accident. PLoS ONE. 2014;9(1):e85723 10.1371/journal.pone.0085723 . 24489667 PMC3906013

[37] United Nations Scientific Committee on the Effects of Atomic Radiation (UNSCEAR). UNSCEAR 2006 Report Annex A. Epidemiological Studies of Radiation and Cancer. New York: United Nations; 2008 p. 13–322.

[38] Ron E, Lubin JH, Shore RE, Mabuchi K, Modan B, Pottern LM, et al Thyroid cancer after exposure to external radiation: a pooled analysis of seven studies. Radiat Res. 1995;141(3):259–77. 7871153

[39] Sigurdson AJ, Ronckers CM, Mertens AC, Stovall M, Smith SA, Liu Y, et al Primary thyroid cancer after a first tumour in childhood (the Childhood Cancer Survivor Study): a nested case-control study. Lancet. 2005;365(9476):2014–23. doi: S0140-6736(05)66695-0 [pii]; 10.1016/S0140-6736(05)66695-0 15950715

[40] Veiga LH, Lubin JH, Anderson H, de Vathaire F, Tucker M, Bhatti P, et al A pooled analysis of thyroid cancer incidence following radiotherapy for childhood cancer. Radiat Res. 2012;178(4):365–76. 10.1667/RR2889.1 [pii]. 22857014 PMC3488851

[41] Preston DL, Ron E, Tokuoka S, Funamoto S, Nishi N, Soda M, et al Solid cancer incidence in atomic bomb survivors: 1958–1998. Radiat Res. 2007;168(1):1–64. . 17722996 10.1667/RR0763.1

[42] Cardis E, Kesminiene A, Ivanov V, Malakhova I, Shibata Y, Khrouch V, et al Risk of thyroid cancer after exposure to 131I in childhood. J Natl Cancer Inst. 2005;97(10):724–32. doi: 97/10/724 [pii]; 10.1093/jnci/dji129 15900042

[43] Shore RE, Hildreth N, Dvoretsky P, Andresen E, Moseson M, Pasternack B. Thyroid cancer among persons given X-ray treatment in infancy for an enlarged thymus gland. Am J Epidemiol. 1993;137(10):1068–80. 8317436 10.1093/oxfordjournals.aje.a116610

[44] Lundell M, Hakulinen T, Holm LE. Thyroid cancer after radiotherapy for skin hemangioma in infancy. Radiat Res. 1994;140(3):334–9. 7972685

